# Corrigendum to ‘Effect of transauricular nerve stimulation on perioperative pain: a single-blind, analyser-masked, randomised controlled trial’ (*Br J Anaesth* 2023; 130: 458–76)

**DOI:** 10.1016/j.bja.2024.12.006

**Published:** 2025-01-18

**Authors:** Amour B.U. Patel, Phillip P.W.M. Bibawy, Juri Ibrahim M. Althonayan, Zehra Majeed, Weng L. Gan, Tom E.F. Abbott, Gareth L. Ackland

**Affiliations:** 1 Translational Medicine and Therapeutics, William Harvey Research Institute, Barts and the London School of Medicine and Dentistry, Queen Mary University of London, London, UK; 2 Barts and the London School of Medicine and Dentistry, Queen Mary University of London, London, UK

The authors regret that an error was present in the Methods section of our recent paper. For the section detailing the active intervention, the current in milliamps was reported incorrectly. Each value should have included a decimal point, that is, 1.0 mA and 2.0–6.0 mA.

The corrected text is as follows:

*Active intervention*

*For active stimulation, electrical stimulation (pulse width: 200 μs; frequency: 30 Hz) was initiated at 1.0 mA. Once the participant reported a ‘tingling’ sensation within 20 s of commencing, the current was reduced to a level just below this threshold (2.0–6.0 mA) and continued for 30 min.*

The authors would like to apologise for any inconvenience caused.

